# Inpainting of damaged temple murals using edge- and line-guided diffusion patch GAN

**DOI:** 10.3389/frai.2024.1453847

**Published:** 2024-11-06

**Authors:** G. Sumathi, M. Uma Devi

**Affiliations:** Department of Computing Technologies, SRM Institute of Science and Technology, Kattankulathur, Chengalpattu, India

**Keywords:** Inpainting, Temple murals, culture preservation, mural dataset, image restoration, generative adversarial network

## Abstract

Mural paintings are vital cultural expressions, enriching our lives by beautifying spaces, conveying messages, telling stories, and evoking emotions. Ancient temple murals degrade over time due to natural aging, physical damage, etc. Preserving these cultural treasures is challenging. Image inpainting is often used for digital restoration, but existing methods typically overlook naturally degraded areas, using randomly generated binary masks or small, narrow regions for repair. This study proposes a novel architecture to reconstruct large areas of naturally degraded murals, maintaining intrinsic details, avoiding color bias, and preserving artistic excellence. The architecture integrates generative adversarial networks (GANs) and the diffusion model, including a whole structure formation network (WSFN), a semantic color network (SCN), and a diffusion mixture distribution (DIMD) discriminator. The WSFN uses the original image, a line drawing, and an edge map to capture mural details, which are then texturally inpainted in the SCN using gated convolution for enhanced results. Special attention is given to globally extending the receptive field for large-area inpainting. The model is evaluated using custom-degraded mural images collected from Tamil Nadu temples. Quantitative analysis showed superior results than state-of-the-art methods, with SSIM, MSE, PSNR, and LPIPS values of 0.8853, 0.0021, 29.8826, and 0.0426, respectively.

## 1 Introduction

Murals seen in South Indian temples represent the customs and cultures of many different religions, making them a cultural asset for India. These murals serve as priceless portals for the past, offering profound insights into the beliefs, customs, and daily life of ancient civilizations. These captivating artworks often found adorning the walls of temples, depict a wide array of subjects ranging from mythological narratives to historical events and religious rituals. Through their intricate details and vibrant colors, ancient murals enable us to understand the cultural, social, and artistic contexts of the time. Moreover, murals serve as primary sources for interpreting and preserving intangible aspects of culture, such as traditions, folklore, and spiritual practices, which may otherwise be lost to the passage of time. Beyond their historical significance, ancient murals also hold aesthetic value, showcasing the artistic achievements and creative expressions of past civilizations.

However, degradation and damage afflict these murals, threatening to erase their historical and cultural significance forever. Factors such as environmental conditions, natural disasters, vandalism, and improper conservation efforts contribute to the deterioration of these artworks. From fading pigments to structural instability, the integrity of these murals is often compromised, necessitating urgent measures for preservation. Preservation efforts for ancient murals are crucial to safeguarding cultural heritage of humanity. Traditional methods such as human repair, environmental controls, and chemical treatments have been employed to halt deterioration and prolong the lifespan of these artworks. While these methods have yielded some success, they often come with limitations. For instance, human repair would be time-consuming, while chemical treatments pose risks to both the artwork and conservators.

In recent years, advances in computer vision and deep learning have opened new possibilities for cultural heritage preservation. By harnessing the power of algorithms and deep learning models, researchers can digitally restore and reconstruct damaged artworks with remarkable precision and visual quality. This interdisciplinary approach combines the expertise of art historians, conservationists, and technologists to address the complex challenges of heritage conservation in the digital age.

Digital preservation techniques, such as image inpainting, offer promising solutions to mitigate damages and restore the integrity of deteriorated murals. Image inpainting involves digitally reconstructing missing or damaged portions of an image based on surrounding visual information. The application of image inpainting in the preservation of ancient murals presents several advantages over traditional methods. It allows for non-invasive restoration, preserving the integrity of the original artwork while effectively repairing damages. Additionally, digital preservation offers scalability, enabling conservation efforts to extend beyond physical constraints. By digitizing ancient murals, scholars, and enthusiasts worldwide can access and study these cultural treasures, fostering greater appreciation and understanding of our shared heritage.

**Figure 1:**
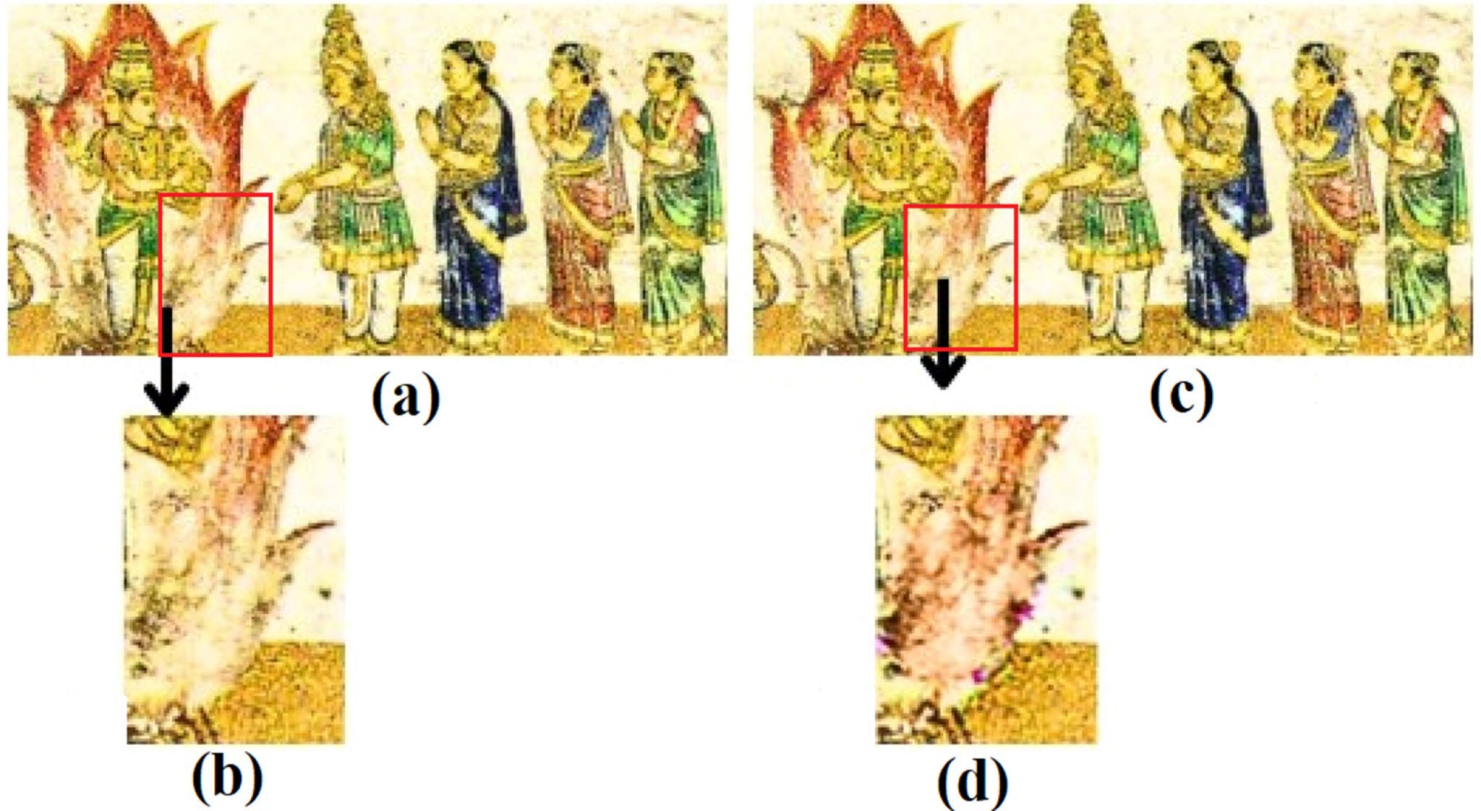
Demonstration of the color bias problem in mural image painting (a) corrupted full mural image; (b) enlarged image showing the area to be inpainted; (c) reconstructed full image; (d) enlarged reconstructed image showcasing the color bias problem in image inpainting.

As different deep convolutional neural networks (DCNN) (Yi et al., 2020; Liu et al., 2019), generative networks have demonstrated improved results in inpainting the natural images found in open datasets such as CelebA (Liu et al., 2015), Places2 (Zhou et al., 2017), and ParisStreetView (Doersch et al., 2015), it is imperative to use deep learning algorithms for the reconstruction of the damaged murals. However, the techniques that have yielded impressive results in natural image inpainting face some difficulties when used on murals. The following are some of the causes of this difficulty:

- Very smooth brushstrokes are used in the mural images, and the textures are largely monotonous.
- The technique of recovery is made difficult by the large and intricate missing areas of the paintings.
- The original, non-damaged image is not available, so the damaged mural image itself serves as the input for reconstruction; hence, the painted image may or may not be remarkably similar to the original.
- Color bias problem. Figure 1 illustrates the color bias problem caused by the mixing of pigments while inpainting murals.

Color bias in image inpainting occurs when the colors in the inpainted region do not blend seamlessly with the surrounding context. This can happen due to improper feature learning or a lack of semantic understanding in the model. This can make the inpainted regions stand out unnaturally, affecting the overall quality and realism of the image. Thus, it is crucial to address this challenge.

**Figure 2:**
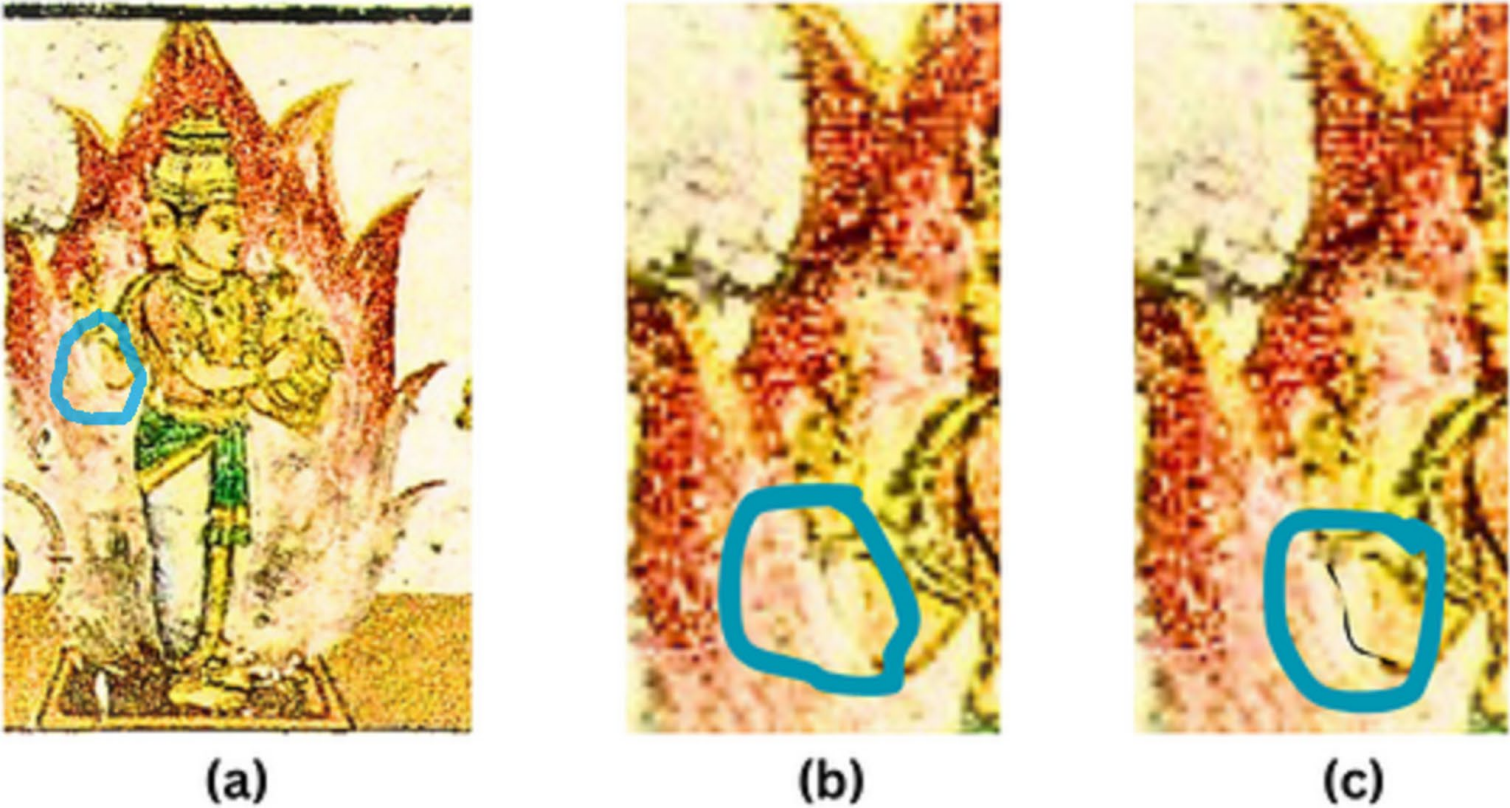
A demonstration highlighting the need of taking lines into account when inpainting a mural: (a) the original image; (b) the inpainting cannot be as accurate as the original without clearly defined boundaries and lines; and (c) the mural inpainting can be more realistic with a line drawing.

Due to the inherent structure and recurring patterns of murals, most of the current algorithms using CNN and GAN are unable to perform inpainting as effectively as they do for natural images, though the inspirations come from these factors (Wu et al., 2023). As elaborate designs of murals are delineated by edge lines, as these lines deteriorate, so do the paintings. If the reconstruction is based on textures alone, as shown in Figure 2, these deteriorating lines do not appear correctly in the reconstruction. Thus, according to the theory put forward in Nazeri et al. (2019), the image inpainting process can be thought of as a two-phase process, with the first phase dealing with structural reconstruction and the second phase dealing with the textural reconstruction of the damaged mural. Due to the complex structures present in mural paintings, focusing just on edge maps is not the best option. Thus, for a better structural definition of the mural, in addition to edge maps, the line drawings (Wang et al., 2019; Li et al., 2022) of the murals are also considered for an artistic-level structure definition. As a result, the proposed approach of inpainting the murals is carried out stage-by-stage as pretreatment and occlusion-aware reconstruction, taking into account the structure and texture.

Section 2 discusses the aim and major contribution of this research work. Section 3 deals with related works. Section 4 explains the materials and methods used in this work. It clearly examines the dataset used and the proposed work in detail. Section 4 presents the experimentation followed by the quantitative and qualitative evaluation of the work. Section 6 discusses the advantages and limitations of the proposed work. The conclusion and future work are illustrated in Section 7.

## 2 Research aim

The existing research studies on the restoration of damaged murals do not consider naturally damaged regions for reconstruction. Instead, damages are recreated digitally through mask-generation algorithms (Ciortan et al., 2021). In some cases, if natural damages are considered, the region of damage is very small (Sun et al., 2022). However, the proposed research work restores damaged murals by considering naturally damaged images. To address the issue of structural blur and large-area filling, the damaged image is rebuilt with the aid of edge and line drawings, guided by the differentiable histogram loss that can substantially improve the damaged mural. Thus, the proposed work aims to reconstruct the damaged portions of ancient murals as a two-step process, by developing a two-stage GAN model that consists of two generators. The first generator network restores the structural portions of the damaged murals with the help of an edge map and line drawings. The second generator network restores the textural portions of the damaged murals and addresses the color bias issue by using the coherence histogram loss function.

### 2.1 Major contribution

This study recommends a mural inpainting approach that is driven by the edge and line link after examining contemporary techniques and understanding the common concerns with mural inpainting. The major contributions of this study are as follows:

- To address the issue of structural blur and big area filling, the damaged image is rebuilt with the aid of an edge map and line drawing for structural reconstruction.
- Color bias issue is addressed through an enhanced coherence histogram loss.
- A novel diffusion-induced mixture distribution (DIMD) discriminator is used to enhance the performance of the generator by incorporating a diffusion process that introduces noise, improving data heterogeneity, and ensuring more accurate structural and color inpainting.
- A custom dataset consisting of damaged ancient temple murals is collected exclusively for this study by visiting many temples across various places in Tamil Nadu, India.

## 3 Related works

### 3.1 Image inpainting

Great strides have been made in image inpainting, and at this point, realistic visuals are being produced that are as close to reality as possible. The standard procedure for inpainting consists of two steps: step 1 seeks to locate the afflicted areas, and step 2 seeks to fill those voids with matching patches. Thus, the effectiveness of inpainting hinges on the ability to precisely mark the areas of the defect and obtain a patch with an identical image. Although this is a straightforward copy-and-paste procedure, it takes some time to look for similar picture patches. As a result, both manual and automated methods can be used to identify related patches.

#### 3.1.1 Traditional methods

Geometrical and patch-based approaches are used to solve inpainting challenges. Differential equations are the foundation mathematics of geometrical approaches. The exterior contents of the hole are transmitted inside it using differentiation. The patch match approach involves using statistical calculations to match the optimum texture for the hole based on the data from the surrounding pixels. Patch match is faster than both options, but the outcome of the inpainting is dependent on the nearby texture (Barnes et al., 2009). As traditional inpainting methods lack knowledge of the image, they cannot produce inpainting that is as close to reality as alternative methods. The solid shape mask can be filled well using patches and geometric shapes, but larger, irregularly shaped holes are more difficult to handle.

#### 3.1.2 Learning-based methods

More recently, data-driven deep learning-based inpainting techniques have improved accuracy as they can inpaint with the image clarity because these networks have a strong understanding of both the local fine textures and the overall image. CNN and GAN are the most used neural network architecture for inpainting. The first GAN for the inpainting job is proposed in Pathak et al. (2016), and because the network is completely connected channel-wise, the network fully comprehends the context of the image. The current layer was able to understand the feature information from the previous layer as the connections were fully connected, which helped improve understanding of the overall image. The quality of the filled image is improved in Yang et al. (2017) by enhancing the contextual encoder from Pathak et al. (2016). With the style transfer approach, which transfers a pixel that resembles the hole to the generator, the local texture details are improved, and the holes have a pleasing appearance.

The cost associated with fully connected layers is addressed in Iizuka et al. (2017), where the concept of deep inpainting evolved. Here, the network using the dilated connection was able to detect the global context of the image as well as the local context using two discriminators. One variation of Iizuka et al. (2017) is given in Demir and Unal (2018) where the method of Iizuka et al. (2017) is boosted using residual learning (He et al., 2016) and patch GAN (Isola et al., 2017). The dilated convolution in Iizuka et al. (2017) was used as an inpainting strategy in various subsequent research. Combining dilated convolution and residual connection results in the creation of a unique dilated residual block. The exactness of the local region is perceived using the matrix labels and the PatchGAN discriminator. As inpainting closely resembles traditional copying and pasting, this study (Yan et al., 2018) attempts to combine the advantages of data and copying, which is achieved using the shift connection layer. This layer attempts to consider the global meaning of the image as well as the local meaning by borrowing information from the nearest neighbors, and the best neighbor is used for filling the hole.

A contextual attention layer is included in DeepFill (Yu et al., 2018), an improved version of Shift-Net (Yan et al., 2018), and it can comprehend the relationship between the features that are missing in the hole and the features that are outside of the hole. It is simple to identify the features of the hole by executing a joining operation on all the characteristics outside the hole. The contribution of each feature in the hole may be determined as each feature is distinguished by its weight. The implicit diversified Markov random field loss functions in generative multi-column convolutional neural networks (GMCNN) (Wang et al., 2018) can improve the local texture details. The improvement occurs as a result of the guiding principle of the created patches, which is to find their closest neighbors from the hole to effectively extract local texture data. Partial convulsions are used in Liu et al. (2018) for handling the uneven holes in multiple regions. As the masks are irregular and at varied spots, the results of the convolution concentrate only on the valid pixels, making the process of filling faster with a controlled setting in the network. Edge map prediction (Nazeri et al., 2019) serving as an inspiration to this work aims to perform the process of inpainting based on the predicted edges of the damaged portions. DeepfillV2 (Yu et al., 2019) is a combined approach of (Wang et al., 2018; Liu et al., 2018; Nazeri et al., 2019) where the concept of gated convolution is introduced that makes the convolution learnable.

Enhanced dynamic memory algorithm is applied in Chen et al. (2023) to capture the local and global features when the missing region is large, which is further followed by a two-step, rough and fine inpainting. To concentrate on inpainting of semantic features (Chen et al., 2024) employed a multiscale feature module to combine features extracted at various scales. It also integrates an attention mechanism to concentrate on the most relevant parts of the image, enhancing the restoration of important features while ignoring less critical areas. A dual-feature encoder is applied in Lian et al. (2024) that integrates structure and texture features to enhance the coherence of contextual semantics and image information. Here, along with the dual encoder, the use of a multiscale receptive field and long–short-term attention provides logical semantic context and removes blurry textures. A residual feature attention network is deployed in Chen et al. (2023) with the aim to improve texture details and reduce artifacts in images with complex and large missing regions. It tries to generate high-quality images by enhancing dense and multiscale feature extraction and optimizing the loss functions.

### 3.2 Mural inpainting

The use of the deep convolutional neural network (DCNN) to determine the age of an artwork is one of the earliest applications of the technology proposed in Zou et al. (2014) and Li et al. (2018). A methodical strategy for identifying scratches and poor coloration has been developed with a focus on the improvement of the color fading and scratches seen in Thailand frescoes (Jaidilert and Farooque, 2018) murals. Here, they have decided on a fundamentally grouped, seed-based technique of region-growing. By combining all the pixels with comparable features, the nearby pixels that are identical to the initialized seeds continue to expand. Once the pixel and seed do not match, the growth stops.

To handle the larger missing regions, an auto-encoder-based methodology is proposed in Song et al. (2020), where the dilated convolutions are utilized for the reconstruction. Inpainting concentrating on the structural aspects is proposed in Ciortan et al. (2021), where the inpainting is done considering the artistic method of coloring. The entire process is carried out step by step in the same way an artist does a painting. Here, the learning is for the edge and colors upon which the inpainting is carried out. Line drawing-guided inpainting is carried out in Wang et al. (2019), in which the inpainting patches are constructed by the combination of multiple patches, and the selection of multiple patches happens using the sparse model construction.

A stroke-like mask generation strategy is proposed in Wang et al. (2020) from which a simulated image is constructed, which then inputted to the partial convolution network was able to generate different types of irregular images and the guiding principle for restoring the original image was two phases. Only the relevant pixels are considered for inpainting, due to the joint predictive filtering and generative network (JPGNet), which combines the filtering and generative approaches. As the resolution and greater hole regions of the original paintings make them unsuitable for training, a data augmentation strategy is suggested in Guo et al. (2021) to improve the quality of the training samples with higher resolution photographs. Chen et al. (2019) and Wang et al. (2021) suggest the use of the partial convolution technique to inpaint Dunhuang and Thanka murals, respectively. In spite of the promising results of gated convolution in inpainting Thanka murals, to overcome the issues like blurring and limited perceptual fields (Jia et al., 2023) used an edge-assisted feature component that impacts the edge details to enhance the texture portions and a self-attention-based local refine module that obtains the long-range relationships to improve the perceptual field. However, the method struggles with larger damaged portions due to the complexity of the Thanka structures. In addition, the model does not consider naturally degraded images. Original images are artificially damaged.

To restore Dunhuang murals (Xu et al., 2023) includes a combination of deformable convolution and CycleGAN to improve mural image inpainting. This combination improves feature extraction and color accuracy, making the restored images look very much like the original murals. However, the method relies heavily on artificially damaged copies leading to randomness. A parallel dual convolutional feature extraction generator along with a ternary heterogeneous joint discriminator is deployed in Ren et al. (2024) to extract detailed features at various scales, ensuring that fine-grained details are accurate. Here, damages are induced into the original images by using a publicly available mask dataset. The model suffers to restore larger damaged areas due to limited semantic information and computational constraints. To reduce information loss and capture semantic details, a dual encoder model that leverages gated encoding is utilized in Sun et al. (2024). A contextual feature aggregation module ensures consistency in the restored image, while a color loss function maintains color harmony with the surrounding areas. The model also struggles to accurately restore details in real damaged murals.

Based on this survey, some issues are quite visible in image inpainting, both at the mural level and in general inpainting. The common issue that is present with regard to filling the larger holes semantically and filling the structure appropriately based on the lines present in the murals. With an understanding of these, a lot of improvement is still required in inpainting, leading to a higher scope of research on this topic.

## 4 Materials and methods

### 4.1 Dataset

**Figure 3:**
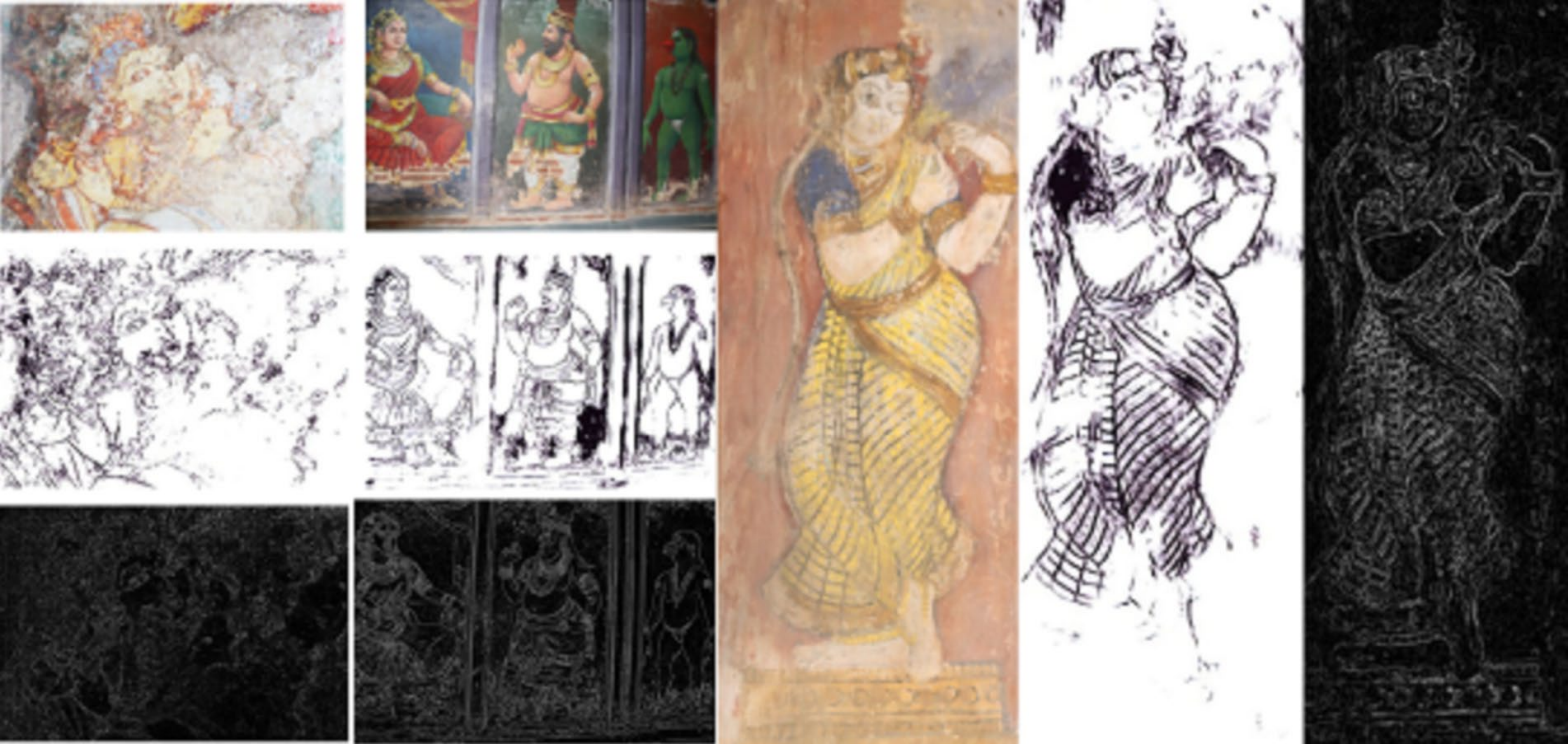
Mural paintings and their corresponding line drawing and edge map in the dataset.

The proposed work is evaluated using a unique dataset that is exclusively collected for this work. The dataset consists of images of damaged custom murals. These mural images are gathered by traveling to the various ancient temples such as Ramaswamy Temple in Kumbakonam, Brihadeeshwarar temple in Tanjore, Kapardeeswarar temple in Thiruvalanchuzhi, Thiyagaraja Swamy temple in Thiruvarur, Sarabeswarar Temple in Thirubuvanam, and Kailasanathar Temple in Kanchipuram, in Tamil Nadu, India. These images are taken using a Canon EOS 200D camera. In addition, certain images of degraded murals are downloaded from https://www.tagavalaatruppadai.in/, which is an official website of the Tamil Nadu Archeology Department. These images consist of the degraded mural paintings from Azhagarkovil, Konerirajapuram, Patteeswaram, and Ramanathapuram. A total of 638 mural images were gathered. As the gathered images are very small in number, these images are preprocessed and augmented as mentioned in Section 4.2. As a result of augmentation, the dataset size is increased to 2,300 degraded images. Furthermore, the dataset is extended to include images of the line drawings and edge maps. DexiNed (Poma et al., 2020) and Canny Edge Detector (Nazeri et al., 2019) were used for the generation of the line drawings and edge maps. Figure 3 presents some of the edge maps and line drawings of the images from the dataset.

### 4.2 Data preprocessing

The gathered mural images are in different sizes. Hence, they are resized to a common size of 512 × 512. As the images are digitally captured, they might contain noise. Hence, the images are denoised using a bilateral filter as it smooths out the noise without blurring the edges, making it ideal for the reconstruction task, thereby maintaining the integrity of the structural details of the mural. After denoising, the images are passed through the Sobel–Feldman filter for sharpening the edges. This filter enhances the edges by emphasizing areas of high contrast, thus making it easier for the network to learn and reconstruct the structural elements accurately. Then, the images are normalized using the mean subtraction and standardization methods. Standardization stabilizes the learning process by centering data around zero and managing the scale of inputs, which helps the model better learn features from damaged areas and improve in detailed reconstruction. As the gathered mural samples are fewer in number, augmentation techniques such as rotation, flipping, scaling, and translation are performed. As a result, the total number of murals increased to 2,300. The dataset was split as 80% for training, 10% for testing, and 10% for validation.

### 4.3 Methods

**Figure 4:**
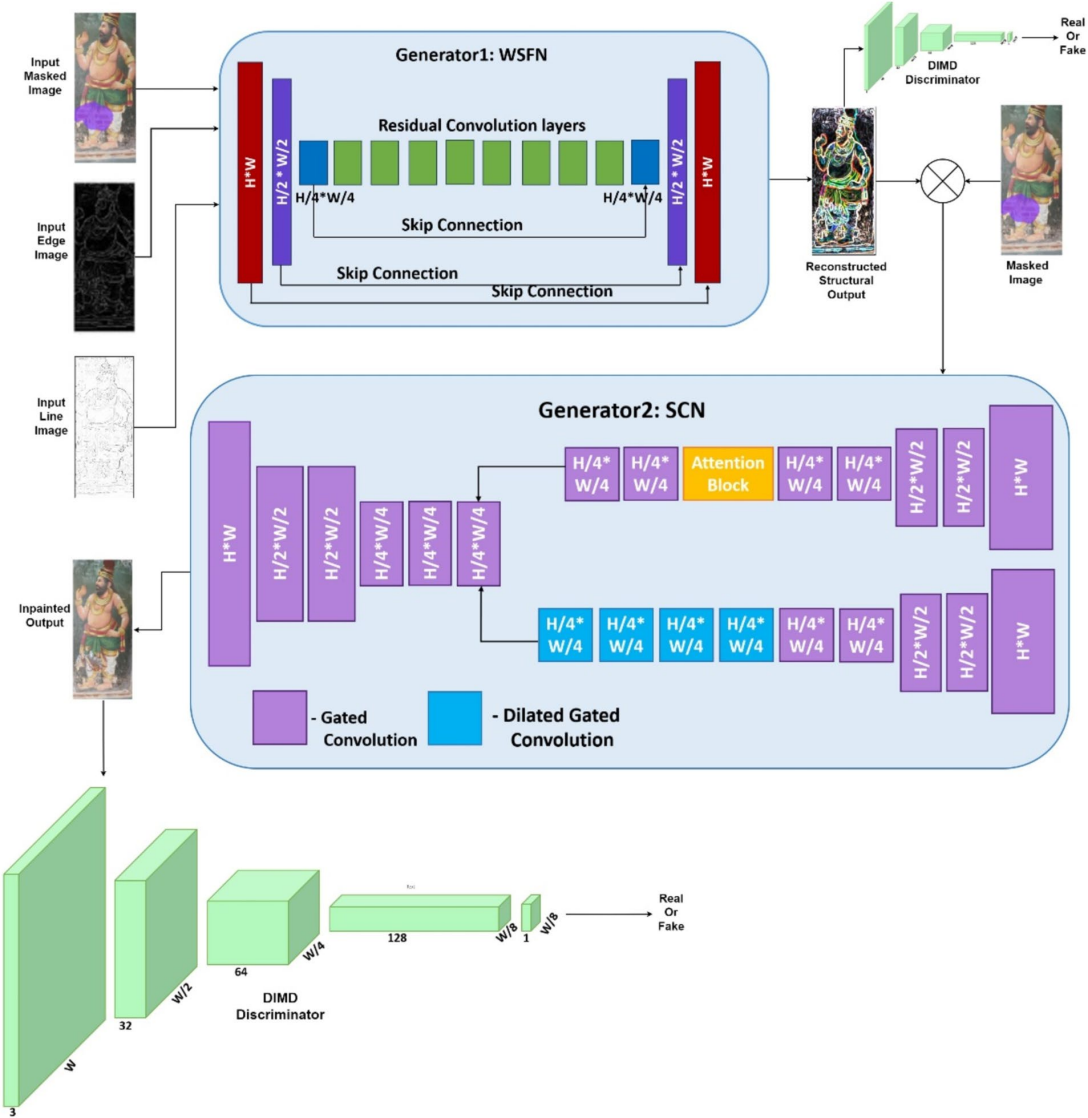
Proposed system architecture.

This proposed method comprises two generative networks, namely the whole structure formation network (WSFN) and the semantic color network (SCN) as shown in Figure 4. WSFN aims to reconstruct the missed structural parts of the mural and SCN takes care of the semantic inpainting, which, in turn, solves the color biasing problem. The reason they are not combined into a single network is due to the fundamentally different tasks they perform—structural reconstruction and texture or color reconstruction. Structural restoration requires precise attention to the geometry and form of the mural, where only the spatial relationships such as the boundaries and contours are relevant. Combining this with texture or color restoration would dilute the focus of the network on the shape and could introduce ambiguities in the structural details. Textural reconstruction demands a different focus—handling variations in pigments, colors, and surface texture. A combined architecture with WSFN would not be able to distinguish the spatial and textural features, leading to confusion in what information to prioritize at different stages of the reconstruction process. Both generators are connected to a single discriminator, as the overall training process lies in reconstructing an image that may be close to the ground truth. Algorithm 1 explains the overall procedure involved in the reconstruction.

#### 4.3.1 Whole structure formation network

The primary goal of this network is to reconstruct the entire structure of the murals, irrespective of the damages. To synthesize such an image, the input to this network is the edge map and line drawing images of the damaged image. Generators perform the process of upsampling and downsampling using the encoder–decoder architecture (Badrinarayanan et al., 2017; Nazeri et al., 2019). The downsampling happens twice so that the image is shrunk to one-fourth of its initial size. This is followed by the eight residual blocks that perform dilated convolutions with a factor of 2, and finally, the decoders up sample the images to their original size. Skip layers are incorporated into the network to gain an understanding of the low-level, multiscale details. This detail is in the form of color information that gets evolved through the skip layers, and hence, the color difference can be easily grasped by the network. The global generation is taken care of by this network as they are directly involved in the calculation of loss values. The WSFN-created synthesized image makes an effort to produce every pixel backward.

For the given original input image, their edge and line drawing combined map is generated by the generator as $WSFN_{\mathrm{Output}}$ . To generate the $WSFN_{\mathrm{Output}}$ the required inputs are the damaged image in RGB format, $\mathrm{Imag}e_{RGB}$ , the corresponding line drawing, $\mathrm{Imag}e_{\mathrm{Line}}$ and edge map, $\mathrm{Imag}e_{\mathrm{Edge}}$ . Let M be the binary mask which is a precondition that mentions 1 for missing regions and 0 for known regions. To focus on the region to be restored, M is applied to the inputs as mentioned in Equations 1–3:


(1) $$\mathrm{Imag}e_{\mathrm{MaskedRGB}=}\mathrm{Imag}e_{RGB}\Theta \left(1-M\right)+M$$

(2) $$\mathrm{Imag}e_{E\mathrm{dgemask}}=\mathrm{Imag}e_{\mathrm{Edge}}\Theta \left(1-M\right)+M$$

(3) $$\mathrm{Imag}e_{\mathrm{Linemask}}=\mathrm{Imag}e_{\mathrm{Line}}\Theta \left(1-M\right)+M$$

Thus, WSFN predicts the line and edge combined image as shown in Equation 4


(4) $$\begin{array}{l} WSFN_{\mathrm{Output}}=\mathrm{generator}1(\mathrm{Imag}e_{\mathrm{MaskedRGB},}\\ \;\mathrm{Imag}e_{\mathrm{Edgemask}},\mathrm{Imag}e_{\mathrm{Linemask}},M) \end{array}$$

For the identification of $WSFN_{\mathrm{Output}}$ to be real or fake, $\mathrm{Imag}e_{\mathrm{Linepred}}$ and $\mathrm{Imag}e_{\mathrm{Edgepred}}$ are given as input to the discriminator. Thus, the network training includes the feature matching loss that is very much similar to the perceptual loss and that is defined under section 4.3.4 as this perceptual loss is modified to include the feature matching as well as the style loss.

#### 4.3.2 Semantic color network

The focus here is on adjusting the pixel values in the missing region by the knowledge gained about the entire image to determine what type of pixel needs to be filled in to make the picture look realistic. This sole goal of the network is not to modify the input image. The residual network that calculates the WSFN’s residual values is essentially this one.

This residual block aims to construct the image painting task with user guidance. It is mentioned as user guidance because the edge map and line drawing have been fed inside the mask as conditional channels. This information from WSFN is expected to traverse across the network, irrespective of how deep the neural network goes. Different channel information should not be combined into a single layer as the network progresses. To address this, a combined approach of gated convolution and residual structure is taken in this study. Gated convolution can learn the features separately for each channel and for every spatial location without them getting combined into a single mask image as the network progresses. By doing this, it is ensured that the features are chosen in accordance with the semantic data of each channel. This makes sure that feature learning is not limited to background and mask.

Therefore, SCN incorporates this gated convolution (Nazeri et al., 2019). In traditional convolution, the entire image is treated uniformly, which can lead to a blending of features that might not be relevant or appropriate for the specific context. Gated convolution, however, introduces learnable soft masks that adaptively control the influence of different regions of the image. These soft masks are learned during training and are applied dynamically, which means the network can learn to suppress or enhance certain features based on their relevance to the inpainting task. This ensures that the colors and textures used to fill in missing areas are more consistent with the surrounding context. Gated convolution performs a form of feature selection at each spatial location. By applying a gate, it can decide which features to pass through and which to suppress. This is crucial for inpainting, where the target is to reconstruct the missing part in a way that is coherent with the remaining parts of the image. For instance, if a particular color is dominant in the surrounding area, the gate can allow features related to that color to pass through, while suppressing features that introduce conflicting colors.

In standard convolutional networks, deeper layers may combine features in a way that results in the loss of specific information, such as color, resulting in mismatched colors in the inpainted area. Gated convolution prevents this by processing features from different channels and spatial locations independently before selective combination, preserving accurate color information.

Context-aware inpainting is also incorporated by gated convolution. It enhances the ability of the network to understand and incorporate context. This is important because the correct color for a missing region often depends on the surrounding content. Gated convolution allows focusing on the relevant context when deciding what color to use in the inpainted area. By dynamically adjusting the contribution of different features based on the context, gated convolution reduces the likelihood of introducing colors that clash with the existing image, thereby reducing color bias.

Hence, a gated convolution network is used in SCN, as mentioned in Equations 5–7.


(5) $$\mathrm{softgat}e_{x,y}=\mathrm{\Sigma \Sigma con}v_{1}.\mathrm{Image}$$

(6) $$\mathrm{featur}e_{x,y}=\mathrm{\Sigma \Sigma con}v_{2}.\mathrm{Image}$$

(7) $$\mathrm{gatedcon}v_{x,y}=\phi \left(\mathrm{featur}e_{x,y}\right)\;\Theta \;\sigma \left(\mathrm{softgat}e_{x,y}\right)$$

In Equation 5, $\mathrm{softgat}e_{x,y}$ represents the soft gating mechanism, where a gating function is learned at every pixel (x, y). Here, the image is passed through the first convolution layer, $\mathrm{con}v_{1}$ which generates a mask that acts like a gate. The sum of all pixels is computed to produce the soft gate values. This soft gate controls how much information at each pixel should pass through. Equation 6 computes the features at each pixel (x, y) by applying another convolution operation, $\mathrm{con}v_{2}$ to the image. These features represent the details the network learns about the texture or color at each pixel in the image. Equation 7 is the final gated convolution equation, where *ϕ* is the activation function applied to the extracted features, and *σ* is the sigmoid activation applied to the soft gate. *Θ* denotes element-wise multiplication. The multiplication of the soft gate and the features allows the network to decide which features should be used at each pixel based on the gating mechanism. This ensures that the reconstruction of texture is guided by the knowledge gained about the entire image while respecting the semantic boundaries defined by the structural information.

Thus, to achieve the task of semantic color filling, SCN takes the concatenation of the WSFN output image along with the masked input. SCN is currently completely aware of the structural pattern of the mural, as this has been well restored using the WSFN network. So, in SCN, it is expected to just modify the missing regions without altering the entire image. This modification can be done with the assistance of the neighboring pixel by performing processing at the space and time domain level. However, when the missing area is large, only the local pixel computation cannot yield a better color, and hence, the computation is required at the non-local region considering the external connection to the hole. Hence special attention is needed in terms of time and space. Thus, this SCN network includes an attention mechanism that can operate in a non-local way by extending the receptive field to the global, as mentioned in Equation 8.


(8) $$\mathrm{missin}g_{i}=\frac{1}{\mathrm{Norm}\left(x\right)}\underset{\nabla_{j}}{\sum }f\left(x_{i,}x_{j}\right)g\left(x_{j}\right)$$

where $\mathrm{missin}g_{i}$ represents the output value for the missing region at position *i*. This value is computed by aggregating information from other positions *j* in the image. $\mathrm{Norm}\left(x\right)$ is a normalization factor that assures the attention weights sum to 1, making the process stable and preventing the output from being skewed by the magnitude of the attention scores. $\Sigma \nabla_{j}$ is the summation over all possible positions *j* in the image. Essentially, it means that the network considers the entire image when determining the value for the missing pixel at position *i*. The function, $f\left(x_{i},x_{j}\right)$ calculates the similarity between the pixels at position *i* and *j*. The similarity score helps determine how much influence the pixel at position *j* should have on the missing pixel at *i*. The function, $g\left(x_{j}\right)$ represents the pixel value at position *j*. It is weighted by the similarity score $f\left(x_{i},x_{j}\right)$ , and then these weighted values are summed to produce the output for the missing region. The similarity function *f* is Gaussian defined, as shown in Equation 9.


(9) $$\underset{\nabla_{j}}{\sum }f\left(x_{i,}x_{j}\right)=e^{\partial x_{i}^{T}\emptyset_{x_{j}}}$$

where $x_{i}^{T}$ is the transpose of the feature vector at position *i* and $\emptyset_{x_{j}}$ is the transformation of the feature vector at position *j*.

#### 4.3.3 Diffusion-induced mixture distribution discriminator

The procedure to acquire the structural reconstruction and fitting with appropriate colors is taken into account with the help of the WSFN and SCN. However, the quantity of training samples is yet another problem with this data. Noise is introduced as input to the discriminator to increase the data accuracy and heterogeneity of the generator network. Here, the diffusion process that blends the noise is applied to both the original image set and the images produced by the WSFN. The performance of generator can be improved by using the transmitted gradients from the discriminator to update the ability of diffusion process to compute the derivative of the output with respect to the input.

The initial step in GAN training aims to execute structural rebuilding, while the second stage focuses on the suitability of the hues. As the final goal is to simply fill up the holes accurately, regardless of their shape, size, or color, the same discriminator is used in common for both generators. Spectral normalization, convolution, and leaky ReLU are all components of the objective function of patchGAN (Ciortan et al., 2021), which also includes the assignment of high probabilities for the real data and low probability for produced data. Therefore, even with the diffusion-infused PatchGAN, the core goal of discriminator of distinguishing between actual and fraudulent images remains the same.

##### ALGORITHM 1 Algorithm for proposed inpainting process

**Table:** 

**Input:** Original Input Image( $\mathrm{Imag}e_{\mathrm{truth}}$ ), Masked Image $\left(\mathrm{Imag}e_{\mathrm{maskedgRGB}}\right)$ , $\mathrm{Imag}e_{\mathrm{Edge}}$ , ${\mathrm{Image}}_{\mathrm{line}}$ **Output:** Reconstructed Image Step 1: for all images in the training set Step 2: Apply Canny Edge Detector and DexiNed to obtain $\mathrm{Imag}e_{\mathrm{Edge}}$ , $\mathrm{Imag}e_{\mathrm{line}}$ Step 3: Compute $\mathrm{Imag}e_{\mathrm{linepred}}$ and $\mathrm{Imag}e_{\mathrm{Edgepred}}$ $\mathrm{Imag}e_{\mathrm{maskedRGB}=}\left(\mathrm{Imag}e_{RGB}\right)\Theta \left(1-M\right)+M$ $\mathrm{Imag}e_{\mathrm{Edgepred}}=\mathrm{Imag}e_{\mathrm{Edge}}\Theta \left(1-M\right)+M$ $\mathrm{Imag}e_{\mathrm{lineepred}}=\mathrm{Imag}e_{\mathrm{line}}\Theta \left(1-M\right)+M$ Step 4: Obtain the structural image from $WSFN_{\mathrm{Output}\;\mathrm{image}}$ Step 5: Combine manual mask image and $WSFN_{\mathrm{Output}\;\mathrm{image}}$ and feed it into SCN Step 6: Compute gated mask $\mathrm{softgat}e_{x,y=}\mathrm{\Sigma \Sigma con}v_{1}.\mathrm{Image}$ $\mathrm{featur}e_{x,y=}\mathrm{\Sigma \Sigma con}v_{2}.\mathrm{Image}$ $\mathrm{gatedcon}v_{x,y=}\phi (\mathrm{softgat}e_{x,y)}\Theta \sigma \left(\mathrm{featur}e_{x,y}\right)$ Step 7: Compute loss and converge

#### 4.3.4 Loss functions

Every GAN network, as is well known, employs two different kinds of losses: one for the generator and the other for the discriminator. The performance of the discriminator in judging the image may be determined using the discriminator loss, and the generator loss can be used to determine how closely the generated image resembles the truth image.

In the proposed network, the generator loss $loss_{\mathrm{generator}}$ includes the pixelwise L1 construction loss and differential histogram loss (Risser et al., 2017). The perceptual loss is not included because the PatchGAN already gives the patch-level information. Thus, the generator loss is obtained using the equation mentioned in Equation 10.


(10) $$loss_{\mathrm{generator}}=loss_{l1}+loss_{\mathrm{patch}}+loss_{\mathrm{histo}}$$

L1 loss expressed as $loss_{l1}$ is the difference obtained between the original image and the predicted image. This loss function determines how far is the inpainted result from the ground truth. Hence, the lower this loss value the distance between the ground and the prediction is less, and hence, the predicted value is closer to the original one. This is computed as mean absolute error (MAE) as formulated in Equation 11.


(11) $$loss_{l1}=\overset{n}{\underset{i=1}{\sum }}{\left(y_{i}-y_{i}^{p}\right)}^{2}$$

The patch loss produced by the PatchGAN performs a good job of describing the style and substance of the image under consideration, but it does have some instability (Gatys et al., 2016); thus, a new coherent-based histogram loss is added to deal with the issue of color bias. The results in terms of texture mapping were just average with the patchGAN loss. The normalization coloring issue can be solved by combining the L1 loss with the patchGAN loss. The network, however, is unable to fill this as it is unsure of the color that each pixel should represent. This L1 loss and patchGAN loss are not combined because the issue of color biasing is the one that is to be addressed. So, a brand-new loss based on the coherence histogram is presented.

Histogram loss addresses this issue by focusing on the distribution of colors in the image rather than just the pixelwise differences. A histogram shows pixel intensity distribution for each color channel. Comparing histograms of generated and non-damaged regions ensures similar color distribution, reducing discrepancies and maintaining color consistency in reconstructed areas. Unlike pixelwise loss functions that only consider local pixel differences, histogram loss takes into account the global distribution of colors. This helps in capturing long-range dependencies and ensures that color consistency is maintained across the entire image.

In this method, it is assumed that the missing region of the input image will share the properties with other parts of the image. So that the missing region can be filled with some other parts of the image that closely resembles the damaged region. Let I be the image and $I_{\mathrm{miss}}$ be the missing region. Some new data are to be supplied to $I_{\mathrm{miss}}$ so that the output, $\hat{I}$ is obtained. This $\hat{I}$ will currently have much global information, and it tries to optimize the color distribution range. Hence, a solution that maximizes the objective function formulated in Equation 12 is required.


(12) $$\begin{array}{l} \mathrm{coherence}\;\mathrm{histo}\left(\hat{I\;}|I_{\mathrm{miss}}\backslash \mathrm{data}\right)=\underset{p\in \mathrm{filldata}}{\sum }\max_{q\in I_{\mathrm{miss}}\backslash \mathrm{data}}\\ \;\overset{L}{\underset{l=1}{\sum }}w_{l}||\mathrm{Histo}\left(\hat{I\;}\right)-\mathrm{Histo}\left(I_{q}\right)|| \end{array}$$

For each pixel *p* in the region to be filled say, filldata , the method looks for the optimal patch *q* from the undamaged parts of the image, $I_{\mathrm{miss}}\backslash \mathrm{data}$ that can be used to fill the missing region. The inner summation calculates the weighted histogram loss for each layer *l* between the histograms of $\hat{I}$ and $I_{q}$ . The weights $w_{l}$ reflect the similarity between patches. They ensure that patches with more similar color distributions are given more importance during the optimization process.

As a result, the histogram is utilized as the basis for the coherence optimization, which uses the expectation and maximization algorithm. At each iteration, the optimal patch for the complete histogram is updated for each output image and its accompanying histogram, to maximize color similarity, making the model capable of replicating various pigments and handling the diversity in mural coloring. Here, the weights only reflect how similar one patch is to the others. These losses that were indicated are not applied to every training period. The histogram loss is not applicable to the WSFN network because it attempts to maximize color similitude. The patchGAN loss, L1 loss, and coherence histogram loss are only applied during SCN training. SCN seeks to compute loss in the omitted region.

## 5 Experiments and results

### 5.1 Training setup

The output of the SCN is affected by the input of the WSFN because the SCN output is dependent on the output of the WSFN. Inputting an image that is far from the original image to SCN will yield severely inaccurate inpainting, so it is important to feed in the right image of WSFN to SCN. Considering this point, the SCN is not required to start the training when the WSFN starts. Hence, here we adopt a two-stage training: at the first stage, the WSFN starts training, and after 40 epochs of training, the WSFN and DIMD come to converge, and the WSFN generates a good structural image with the integration of line and edge maps. At this stage, the SCN also takes part in the training process, and the discriminator can perform its task easily; hence, the convergence of the SCN happens quickly.

This proposed method is implemented using PyTorch and CUDA 10.1, and the network is trained with 512*512 images with a batch size of 8. The optimization is done using the Adam optimizer with *β*_1_ = 0.9 and *β*_2_ = 0.999 for a total of 60 epochs on four NVIDIA GTX 2080 GPUs. WSFN and SCN are trained separately with a different learning rate until the losses plateau. Then, the training rate is reduced, and the discriminator is set to 0.00001 and trained until convergence. The epoch and batch size were set to 40.8 in the first training stage and 20.8 in the second training stage.

To mitigate the risk of overfitting, several measures were taken during the training process:

- *Data augmentation:* As mentioned, the dataset is expanded through augmentation, which helped introduce more variability and prevent the model from memorizing specific patterns in the limited dataset.
- *Regularization techniques:* Dropout layers are employed in the network, thereby reducing over-reliance on specific neurons and improving the model’s ability to generalize.
- *Validation split:* Train–validation–test split of 80:10:10 is done to carefully monitor the performance of the model on unseen validation data during training. This allows keeping track of how well the model was generalizing beyond the training set.
- *Reduced complexity:* To avoid overfitting on a smaller dataset, a balance between model complexity and training data size was maintained. This helped ensure that the model could learn without becoming too complex to generalize effectively.

### 5.2 State-of-the-art comparison

Here, four cutting-edge methods (Nazeri et al., 2019; Yu et al., 2019; Li et al., 2020; Li et al., 2022)—are compared qualitatively and quantitatively with some of the best-performing image inpainting techniques. Qualitative analysis helps us comprehend the benefits and drawbacks of each strategy by providing a visual representation of the inpainting results of various approaches. A rough understanding of the outcomes about the metrics is provided by quantitative analysis. A study on ablation is also conducted to determine how well the suggested strategy works. The integrated mural dataset is used throughout the entire experiment.

Training DeepFillv2 (Yu et al., 2019), Edge Connect (Nazeri et al., 2019), RFR (Li et al., 2020), and MuralNet (Li et al., 2022) allows for comparison with the four cutting-edge techniques. The Mural-built data to train MuralNet, DeepFillv2, and Edge Connect to conduct a fair comparison. Despite having various approaches, these techniques share many characteristics with the suggested methodology in terms of architecture, structural guidance, and attention mechanism. The comparison based on dimensions is shown in Table 1.

**Table 1:** Comparison of inpainting approaches with various dimensions such as multistage, line guided, edge guided, and attention mechanism.

Method	Multi-stage	Line guided	Attention mechanism	Edge guided
DeepFillv2 (Yu et al., 2019)	Yes	Yes	Yes	No
Edge Connect (Nazeri et al., 2019)	No	Yes	No	Yes
RFR (Li et al., 2020)	No	No	Yes	No
RFA-Net (Chen et al., 2023)	Yes	No	Yes	No
MuralNet (Li et al., 2022)	Yes	Yes	Yes	No
Proposed	Yes	Yes	Yes	Yes

### 5.3 Qualitative comparison

**Figure 5:**
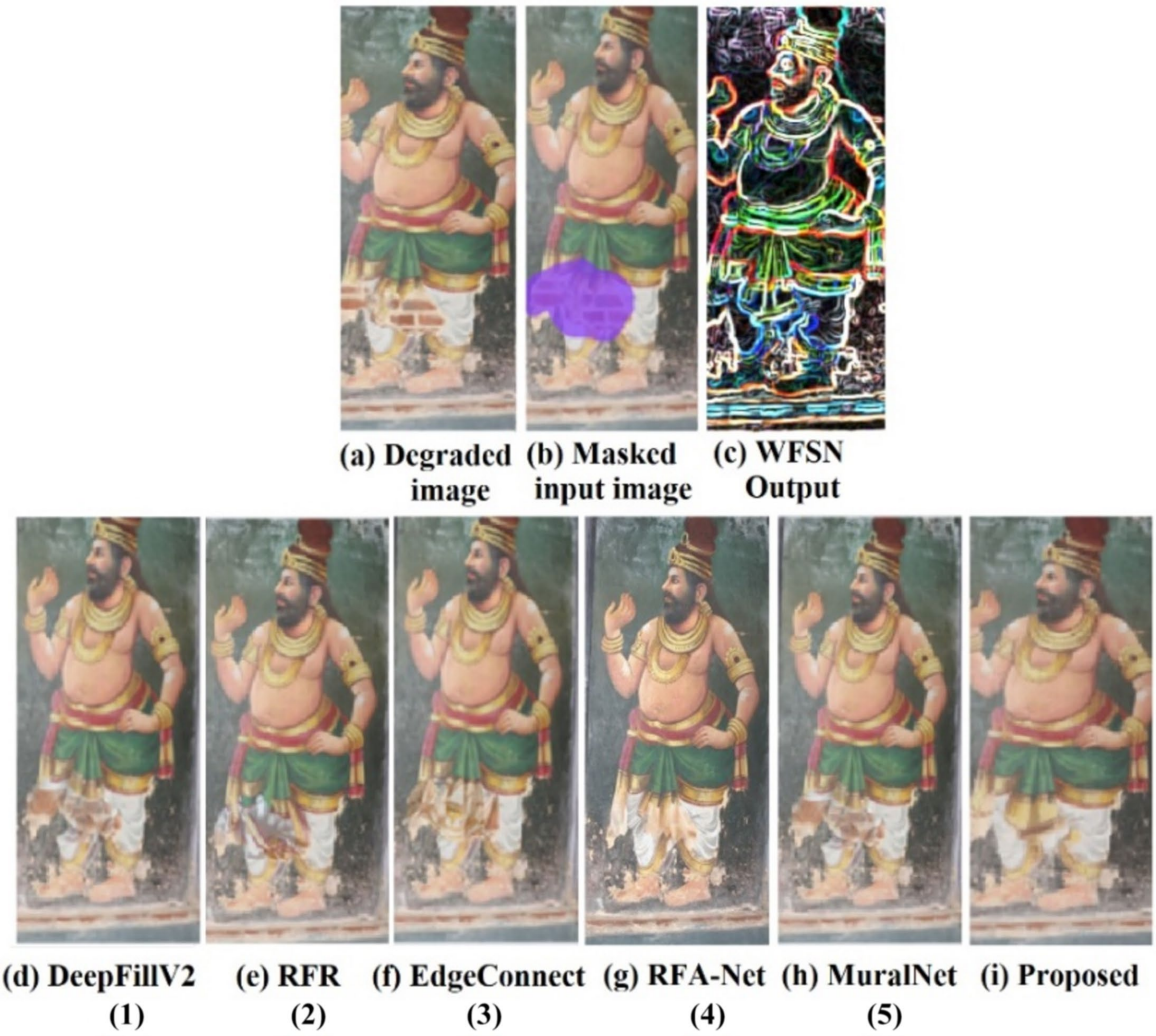
Qualitative analysis of the image from the captured mural of Sarabeswarar temple, Thirubuvanam, which is marked with a free-form larger hole, and the inpainting aims in reconstructing the dhoti to its exact form. (1) Yu et al. (2019), (2) Jia et al. (2023), (3) Nazeri et al. (2019), (4) Nazeri et al. (2019), (5) Li et al. (2022).

**Figure 6:**
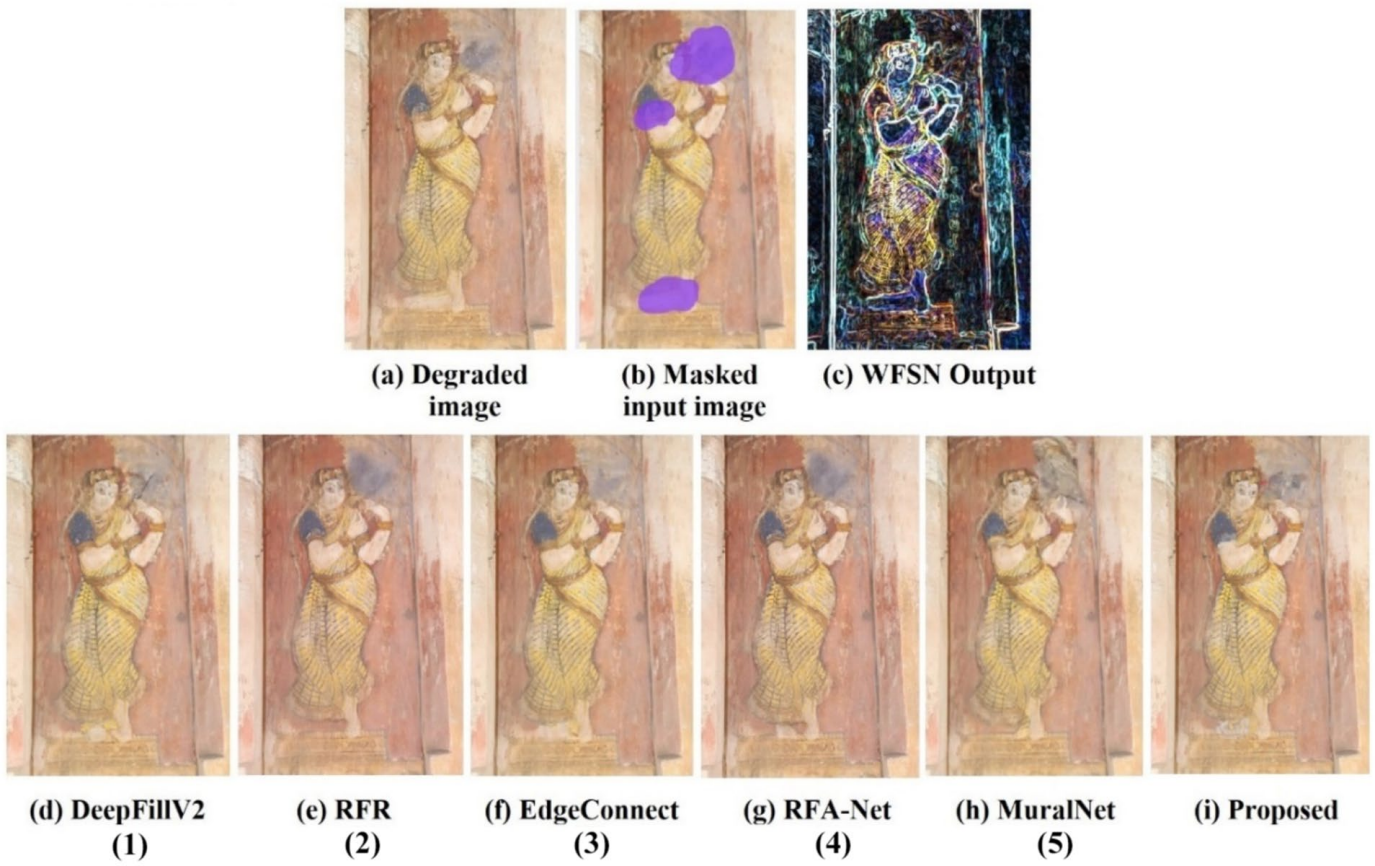
Qualitative analysis of the image from the captured mural of Kapardeeswarar temple in Thiruvalanchuzhi, which is marked with a free form larger and multiple holes. The inpainting aims in reconstructing the eyes and legs. The reconstruction of the eyes of the proposed is better than any other reconstruction of the method. (1) Yu et al. (2019), (2) Jia et al. (2023), (3) Nazeri et al. (2019), (4) Nazeri et al. (2019), (5) Li et al. (2022).

**Figure 7:**
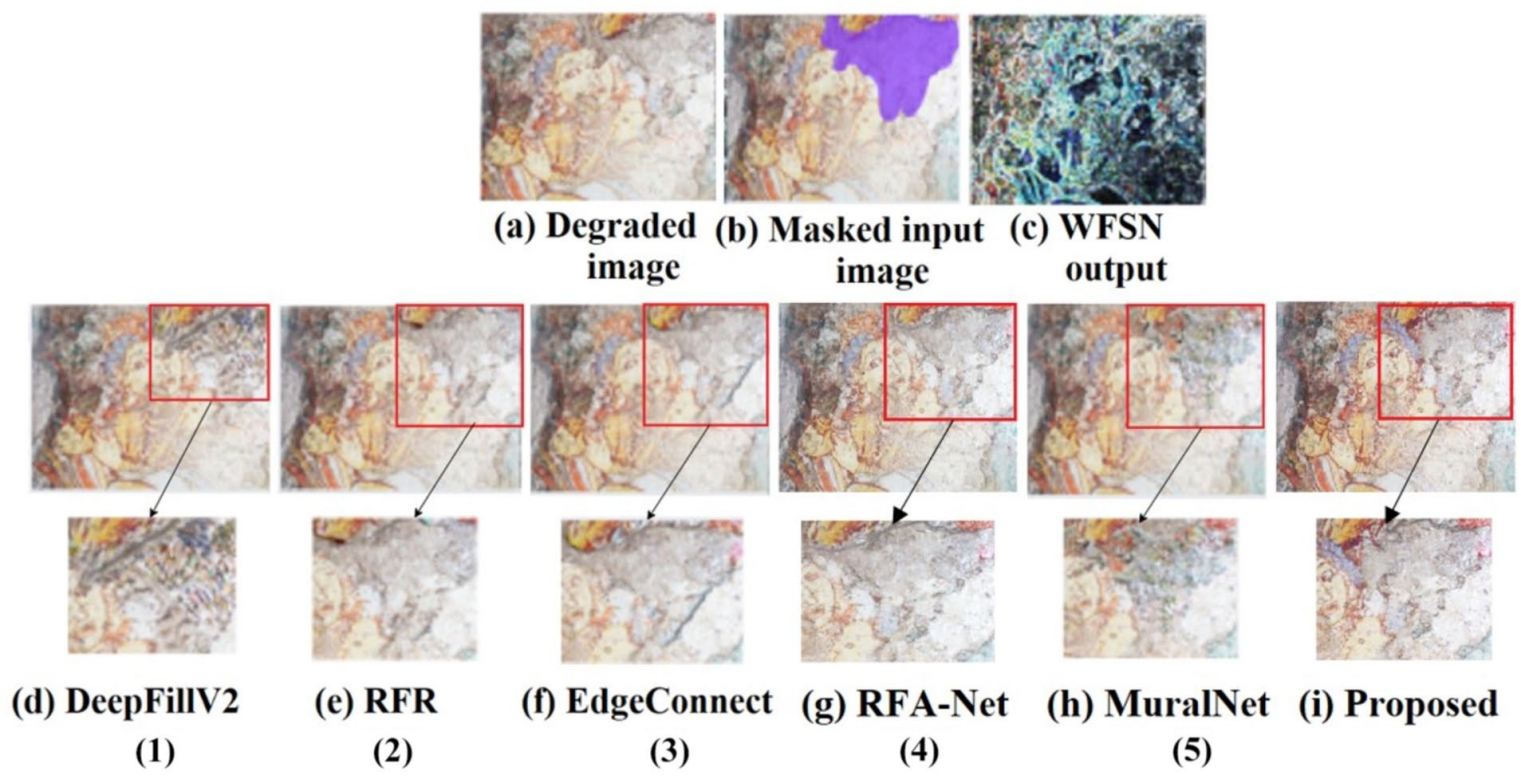
Qualitative analysis of the mural taken from Kanchipuram Kailasanathar temple, marked with a free-form larger hole with inpainting results for both proposed and existing approaches. The inpainted regions are marked in red and are zoomed out for a detailed view (1) Yu et al. (2019), (2) Jia et al. (2023), (3) Nazeri et al. (2019), (4) Nazeri et al. (2019), (5) Li et al. (2022).

The qualitative analysis is done using three images of the dataset, and the results are shown in Figures 5–7. The masks are done manually, and the structure obtained using the WSFN is used for performing the inpainting. As seen in these figures, the structural properties are well restored in the proposed approach, especially the reconstruction of eyes in Figures 6, 7. This is the major variation observed between the proposed approach and the other approaches. Compared with the state-of-the-art, poor performance is observed with the RFR method. This may be due to the absence of line guidance and attention mechanisms.

As far as DeepfillV2 is concerned, the images generated suffered from less color bias as compared to Edge Connect. The performance of Edge Connect in terms of structural reconstruction is comparably good; however, it suffers from a color bias problem. In Figures 6, 7, MuralNet, EdgeConnect, and the proposed method were able to reconstruct the eyes almost identically; however, the color bias in EdgeConnect was higher. MuralNet coloring was comparable to that of DeepFill and EdgeConnect. RFA-Net shows good performance in texture reconstruction but struggles in reconstructing the structures. Again, this may be due to the lack of edge and line guided mechanisms. The proposed method outperformed, as the coloring shows the texture associated as well. Thus, in comparison with the state of the art, the proposed method achieves good structure, texture, and color. Training DeepFillv2 (Yu et al., 2019), RFR (Li et al., 2020), Edge Connect (Nazeri et al., 2019), RFA-Net (Chen et al., 2023), and MuralNet (Li et al., 2022) allows for comparison with the five cutting-edge techniques. The mural-built data are used to train MuralNet, DeepFillv2, and Edge Connect to conduct a fair comparison. Despite having various approaches, these techniques share many characteristics with the suggested methodology in terms of architecture, structural guidance, and attention mechanism.

**Figure 8:**
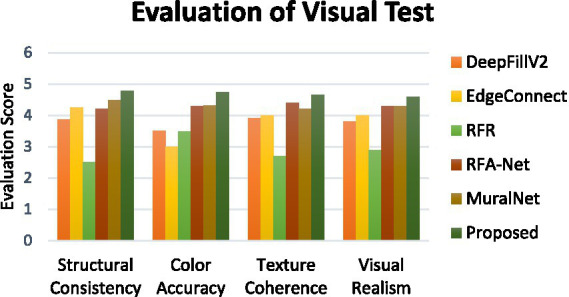
Comparison of evaluation scores of visual test.

To visually evaluate the performance of the model, a real-world testing was conducted. The test involved 30 volunteers as participants. Ten inpainted mural images are chosen for evaluation. The participants were instructed to evaluate the mural outputs from the proposed and existing methods, assigning a score out of five for each factor, such as structural consistency, color accuracy, texture coherence, and visual realism. Figure 8 shows the comparison of the average scores of the proposed and existing methods obtained from the real-world visual test. It is observed that the proposed method performs better by achieving the highest score than other methods.

### 5.4 Quantitative comparison

To comprehend the pixel difference, structural similarity, image quality, and image inpainting that is more similar to human restoration, a quantitative evaluation is carried out. The evaluation metrics MSE (Allen, 1971), SSIM (Wang et al., 2004), PSNR (Hore and Ziou, 2010), and LPIPS (Zhang et al., 2018) can be used to represent them. For the evaluation of these measures, 63 images from the dataset are taken into account.

**Figure 9:**
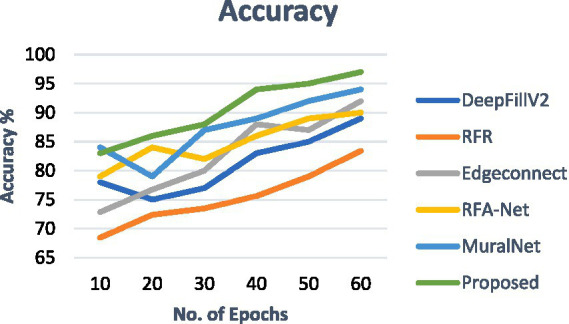
Accuracy comparison of proposed work.

As shown in Table 2, the performance of the proposed system is better in terms of SSIM, MSE, PSNR, and LPIPS. The inpainting effect was fairly good for different masks as well. Irrespective of the type of mask ratio, the PSNR and SSIM values were better. For the PSNR values, the MuralNet and EdgeConnect were very close, and for the SSIM values, DeepfillV2 and RFA-Net were close to each other. Compared with different mask ratios, both at the PSNR and SSIM, the RFR network behaves poorly due to a lack of structural knowledge. In addition, the accuracy comparison of the proposed model with the existing models is shown in Figure 9.

**Table 2:** Quantitative results for the mural inpainting.

Method	SSIM	MSE	PSNR	LPIPS
DeepFillV2 (Yu et al., 2019)	0.7662	0.0063	22.6394	0.1529
EdgeConnect (Nazeri et al., 2019)	0.8258	0.0048	25.1153	0.1274
RFR (Jia et al., 2023)	0.6724	0.0179	21.7935	0.2738
RFA-Net (Chen et al., 2023)	0.7851	0.0057	23.4522	0.1462
MuralNet (Li et al., 2022)	0.8349	0.0043	25.6361	0.1085
Proposed	0.8853	0.0021	29.8826	0.0426

## 6 Discussion

In this study, we have proposed an architecture that considers the problem of color biasing and structure reconstruction for the murals. Given a damaged mural, we formulated the problem of the reconstruction as structure reconstruction and semantic color correction and used GAN-based models to minimize the histogram estimation. Experimental analysis in terms of qualitative and quantitative results shows that our approach can generate very good inpainting results, even for the intricate details of the image. Specifically, the use of combined line and edge drawings has enhanced the structural details of the input image, resulting in a better reconstruction. The comparative analysis also shows the superiority of our approach to the mural damages and, hence, a competitive inpainting performance with state-of-the-art models.

Though the experimentations yield impressive results, it suffers from a few limitations. The damaged area is manually masked and given as input. Automatic damage detection is not done here. To do so, it involves the classification and identification of various types of damages, such as cracks, flakes, scratches, and discoloration. This requires a separate network to detect and classify the damages. Integrating this network will elevate the computational complexity of the proposed model. Then, the model struggles to inpaint multiple damages simultaneously as shown in Figure 6, where trying to inpaint the eyes, hand, and foot portions simultaneously does not provide good restoration results. Only the eye portion is restored, whereas the others are not perfect. This may be due to the following reasons. In WSFN, the skip connections help preserve low-level details, but combining information from multiple damaged regions can become challenging, especially if regions overlap or are close together. In SCN, the contextual information provided by gated convolution is insufficient to address the global context when multiple regions are involved. The attention mechanism aims to aggregate information globally, but if multiple damaged areas require different types of contextual information, the attention mechanism might struggle to balance these needs effectively. To address this issue, the skip connections in WSFN can be enhanced with multi-level skip connections or contextual skip connections and context-aware techniques such as feature pyramid networks can be used in SCN, thus providing scope for further research.

### 6.1 Ablation study

**Figure 10:**
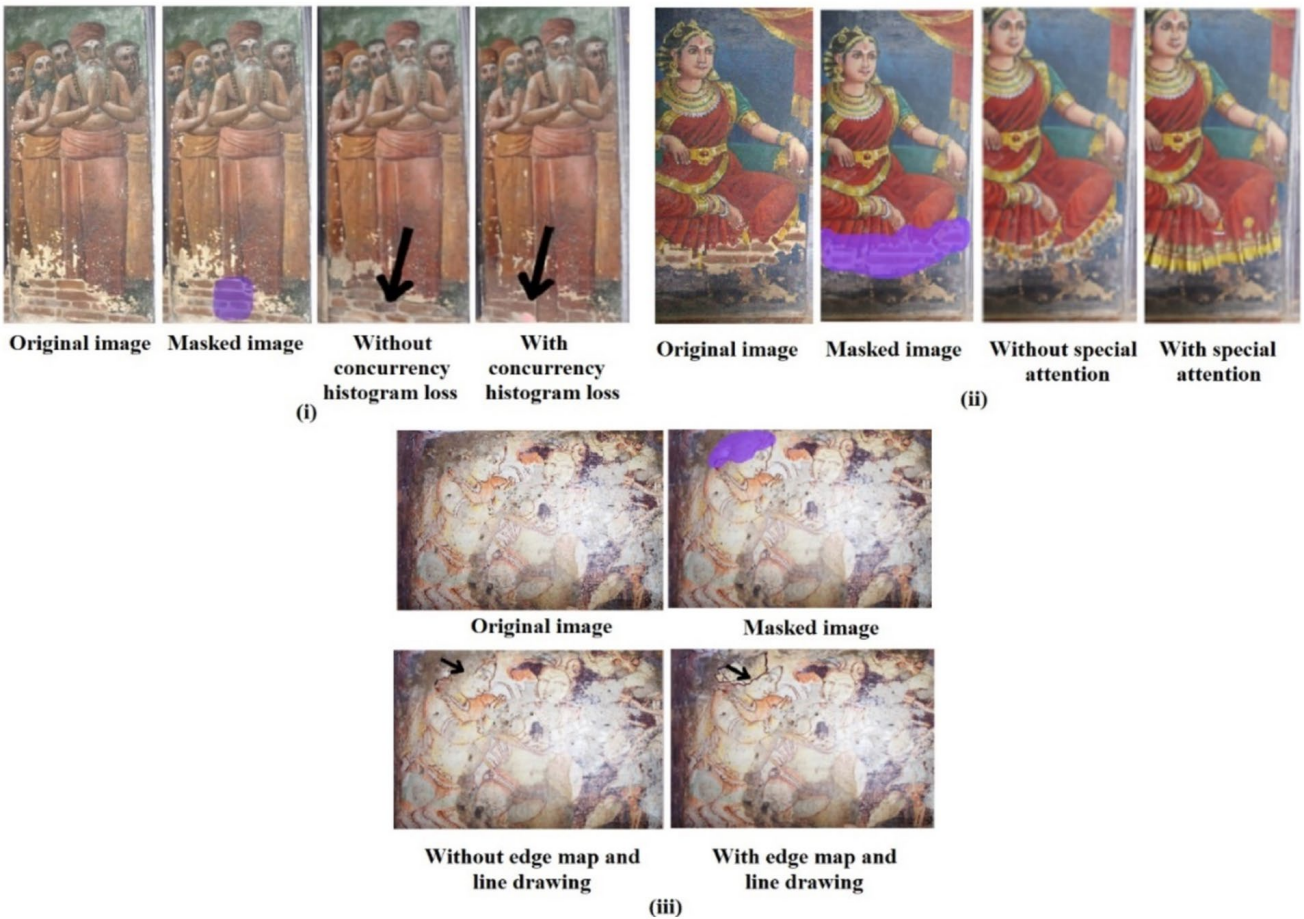
Ablation study of (i) concurrency histogram loss; (ii) special attention mechanism; (iii) edge map and line drawing.

As several methodologies are used, the presented results are comparatively strong when compared to the state of the art. The importance of coherent histogram loss, WSFN with edge map and line drawing and SCN, the attention matrix, and diffusion inside the PatchGAN modules are analyzed by removing them one at a time. This is done to assess their inclusion and their inpainting effect to demonstrate the effectiveness of the suggested methodology. As clearly shown in Figure 10 (i), the inclusion of histogram loss has significantly improved the color biasing that is observed about the reconstruction without the histogram loss, and a similar observation is made with the inclusion of the attention layer. The attention layer in the SCN network thus ensured the semantic color reconstruction as viewed in Figure 10 (ii) the definitive structure defined with the inclusion of WSFN ensures that structural variations are made about the damaged image. The importance of combining the edge map and line drawing along with the input image is visually clear from Figure 10. From Figure 10 (iii) it is evident that the original image provides a global context for the missing region, the line drawing captures fine structural details, ensuring that intricate lines or shapes within the mural are reconstructed and the edge map helps preserve sharp transitions and edges, particularly around damaged areas, ensuring smoother boundary recovery. Without these components, a generator relying solely on convolutional layers would lack the explicit structural and edge information necessary to reconstruct large missing regions accurately, leading to less precise results, especially in complex mural images with high variability in style, texture, and color. In essence, the approach developed aimed to address the problems with color biasing and plug the gaps with structural knowledge. This indicates unequivocally how the addition of these modules to the system has facilitated structural inpainting.

## 7 Conclusion

This work proposed a structure-guided inpainting method for handling larger missing regions in the murals. A novel pipeline is built as a multistage network where specific needs are satisfied with the inclusion of various modules. The main part of the generator aims to reconstruct the exact structure of the damaged image, which, when painted semantically, can yield good results. The combination of the line drawing and edge map by the WSFN network reconstructed the structure by repairing the missing regions in the line drawing and edge map. The SCN network, guided by the coherency histogram loss, can resolve the issues with color bias. The diffusion inside the PatchGAN and coherency histogram loss were used for the first time in the image inpainting of murals. Moreover, mural images from several temples in India were collected, and a database was built. A quantitative and qualitative assessment of the proposed approach shows the superiority of this approach over state-of-the-art methods. The efficacy of each of the concepts in the proposed system has been well-studied using ablation experimentation. Though the results are impressive compared to the state of the art, the inpainting results can be further improved. To enhance the inpainting results in multiple larger regions, stable diffusion and relative approaches need to be fine-tuned.

The presented work uses manual selection of damaged regions for inpainting. The future work will focus on the automatic detection of damaged regions to inpaint. In addition, it is planned to widely extend the dataset with murals from various regions and historical periods to increase the generalizability of the model. It is devised to investigate how prompt-based techniques can be adapted to this mural restoration task in the near future. This could involve incorporating textual descriptions or historical information about the murals to guide the inpainting process, thereby improving the accuracy and fidelity of the restorations. Recent advancements in this technique will be explored to assess its applicability for enhancing the overall restoration quality of the research.

This research can be enhanced further by integrating stable diffusion techniques into the generator networks and improvising the loss functions. The coherence-based histogram loss can be extended by incorporating content awareness by computing histograms not only based on color distribution but also on the content features extracted from deeper layers of a pre-trained network. This leads to better handling of variations in pigments and mural styles. Furthermore, the patchGAN loss can be modified to attention-enhanced PatchGAN Loss, conditioning the discriminator to give higher priority to areas where fine details, such as edges or key design elements, are more important. A stable diffusion process can be integrated with the SCN to iteratively refine the texture and color consistency by denoising the generated image toward more realistic, contextually accurate outputs.

## Data Availability

The original contributions presented in the study are included in the article/supplementary material, further inquiries can be directed to the corresponding author.
